# How Water Content Can Influence the Chemomechanical Properties and Physical Degradation under Aging of Experimental Adhesives

**DOI:** 10.1155/2022/5771341

**Published:** 2022-02-27

**Authors:** Stella Renata Machado Silva Esteves, Daphne Camara Barcellos, Tânia Mara da Silva, Mateus Rodrigues Silva, Tiago Moreira Bastos Campos, Elizabeth Pimentel Rosetti, César Rogério Pucci, Sérgio Eduardo de Paiva Gonçalves

**Affiliations:** ^1^Department of Restorative Dentistry, Institute of Science and Technology of São José dos Campos, Sao Paulo State University (Unesp), São Paulo, Brazil; ^2^Department of Prosthodontics, Federal University of Espírito Santo, Vitória, ES, Brazil; ^3^Anhanguera College & University of São José Dos Campos, São Paulo, Brazil; ^4^Technical Institute of Aeronautics, São Jose dos Campos, São Paulo, Brazil

## Abstract

**Objective:**

To evaluate the physicochemical (sorption (SOR), solubility (SOL), and degree of conversion (DC)) and mechanical (flexural strength (FS), modulus of elasticity (ME), and compressive strength (CS)) properties of adhesives with different water contents (D_2_O).

**Materials and Methods:**

An adhesive was formulated: 55 wt% BisGMA, 45 wt% HEMA, 0.5 wt% camphorquinone, 0.5 wt% EDMAB, and 1.0 wt% DPIHP. D_2_O was added into the adhesives (0 wt%, 10 wt%, and 16 wt%). DC was monitored through the FTIR. FS, ME, CS, SOR, and SOL were tested. The adhesive samples were aged in deionized water, ethanol, and acetone. Data were submitted to ANOVA and Tukey's tests (5%).

**Results:**

For DC, the 0 wt% group showed a significant reduction (68.09 ± 0.14_A_) compared with the 10 wt% (87.07 ± 0.81_B_) and 16 wt% groups (89.87 ± 0.24_B_); 10 wt% showed the highest FS (MPa) mean values (141.6 ± 6.71_B_) compared with the 0 wt% (109.4 ± 20.5_A_) and 16 wt% (107.8 ± 15.8_A_). For the CS (MPa) and ME (GPa), the 16 wt% showed the lowest mean values (98.8 ± 18.0_B_ and 2.2 ± 0.3_B_, respectively) compared with the 10 wt% and 0 wt%. For the SOR, 16 wt% of water showed the highest mean values and the ethanol showed the lowest mean values of SOL regardless of water content.

**Conclusion:**

The amount of water content and the types of aging solvents significantly affect the adhesive properties.

## 1. Introduction

Dental composites are becoming more popular because of their strength, rapidness, and control of polymerization and aesthetic appearance. However, there is a large percentage of failures in a short period in these restorations [1]. Failures of composite restorations are observed mainly at the dentin/adhesive interface. The factor for the long-term success of the composite restoration is the integrity of the adhesive bond layer with the presence and quality of the hybrid layer [2].

The factors that limit the durability of the adhesive layer are incomplete polymerization, partial infiltration of the adhesive into the demineralized dentin matrix, phase separation, and hydrolytic degradation of the adhesive interface [3].

The moisture of the dentin substrate after acid etching preserves the interfibrillar spaces of the collagen network for infiltration of the adhesive and builds a homogeneous hybrid layer [4]. However, clinically, there is a difficulty in controlling the humidity. Excess water forms water blisters inside the adhesive, and phase separation at the adhesive/dentin interface has appeared as a new type of bond defect [5], dilutes the hydrophilic adhesive monomer and plasticizer polymer, and accelerates degradation of the adhesive interface [6].

Ye and colleagues [7] developed a ternary phase diagram of a dentin adhesive model composed of BisGMA (bisphenol A-glycidyl methacrylate), HEMA (hydroxyethyl methacrylate), and water. This ternary phase diagram provides valuable quantities of information regarding miscibility, distribution ratio, and phase portioning of the three components [7]. The hydrophilic-rich phase is composed primarily of water and HEMA. Under clinical conditions, the amount of water in the composition of the adhesive system should probably affect the adhesive stability, can limit the polymerization of the hydrophilic-rich phase, and leach HEMA to the surrounding tissues, inducing apoptosis [8], interfering in DNA synthesis. With the production of reactive oxygen species [9] and the expression of type I collagen by gingival fibroblasts, inhibition of mineral formation of dentin can occur [10]. In addition to the water from the hydrated dentin through the osmotic process, which may be increased by the presence of pulpal pressure, the dental adhesive can also present water sorption from the wet oral environment [11].

In the oral environment, the composite restorations are continuously exposed to chemical agents found in saliva, food, and drinks. These agents, when associated with temperature changes and dynamic load during chewing, may affect the long-term properties of a composite in the mouth [12]. Some organic solvents have the potential to damage the polymer structure of a composite [13]. It has been reported that the immersion in solvents also accelerates the degradation of the material, softens the polymer matrix through plasticization, and facilitates the release of unreacted monomers and degradation products, inducing similar effects already mentioned with regard to the wet environment [14].

Therefore, the objective of the present study was to evaluate the chemomechanical properties and physical changes under aging with different types of solvents of experimental adhesive with different water contents (D_2_O). Thus, the null hypotheses tested were as follows: (1) the model adhesive with different water contents (D_2_O) could not achieve similar results for the chemomechanical and physical properties; (2) the different aging solvents could not induce similar effects in the physical (sorption/solubility) properties of the experimental adhesives.

## 2. Materials and Methods

### 2.1. Model Adhesive Compositions

The model adhesive consisted of HEMA (Sigma-Aldrich, St. Louis, MO, USA) and BisGMA (Sigma-Aldrich, St. Louis, MO, USA) with a mass ratio of 45/55 (HEMA/BisGMA). The photoinitiators used were as follows: 0.5 wt% camphorquinone (CQ) as a hydrophobic photosensitizer, 0.5 wt% ethyl-4-(dimethylamino)benzoate (EDMAB) as a hydrophobic reducing agent, and 1.0 wt% diphenyliodonium hexafluorophosphate (DPIHP) as a hydrophilic coinitiator (all from Sigma-Aldrich, St. Louis, MO, USA). The neat resins were prepared in brown glass vials and stirred for 48 h to form a homogeneous solution [15].

D_2_O (99.9%; Sigma-Aldrich, St. Louis, MO, USA) was added into the neat resins in variable amounts: 0 wt%, 10 wt%, and 16 wt%, based on [5]. These concentrations of D_2_O were added according to a ternary phase diagram [16]. D_2_O (heavy water) was used instead of water to avoid coincidence of the peaks at the spectrum in the FTIR [16].

### 2.2. Degree of Conversion (DC)

The DC was monitored *in situ* with an infrared spectrometer (FTIR/ATR, PerkinElmer, Waltham, MA, USA) with a resolution of 4 cm^−1^.

A volume of 10 *µ*L of the experimental adhesive model was placed on the ATR crystal, and a transparent coverslip was attached with a piece of tape placed on the sample to prevent the evaporation of components [6]. A 20-s-exposure to the LED unit (Demi Light Curing System, Kerr Corporation, USA), at an intensity of 1200 mW/cm^2^, was initiated after the 50 spectra had been recorded. Real-time infrared spectra were continuously recorded for 600 s: before, during, and after light curing [17]. A time-resolved spectrum collector (Spectrum TimeBase, PerkinElmer, MA, USA) was used for continuous and automatic collection of spectra during light curing.

The DC was determined using the following equation, which was based on the intensity band ratios (peak area) before and after light curing (from 1638 cm^−1^ to 1608 cm^−1^) [16] (equation (1)). The DC was carried out in triplicate, and the results were averaged. The rate of polymerization was determined by taking the first derivate of the time vs. DC curve.

### 2.3. Flexural Strength and Modulus of Elasticity

Ten specimens of each group (0 wt%, 10 wt%, and 16 wt% D_2_O) were prepared. The uncured adhesive model was placed on rectangular silicon molds (12 mm length × 2 mm width × 2 mm height) [18], which were covered with a Mylar strip and light cured from the top surface for 20 s (Demi LED Light Curing System) at 3 different positions (right, middle, and left). The bottom surface was also light cured for another 20 s each (right, middle, and left). Specimens were stored in distilled water for 48 h at 37°C to complete the polymerization.

The flexural properties were evaluated using a three-point flexural strength test performed with a universal testing machine (EMIC DL-200 MF, São José dos Pinhais, SP, Brazil), at a crosshead speed of 0.5 mm/min. Flexural strength was obtained by measuring the load at fracture, and the modulus of elasticity was calculated based on the recorded load deflection curves [19].

### 2.4. Compressive Strength

Ten specimens of each group were prepared using a silicon mold (4.0 mm diameter × 8.0 mm height).

The silicon mold was filled in four approximately 2.0 mm thick increments and light cured for 20 s (1200 mW/cm^2^; peak wavelength of 453 nm; LED Light Curing System, Demi Plus, Kerr Corporation, WI, USA) for each layer. The last increment was covered with a Mylar strip and a glass slide and light cured for 20 s. Additional light curing was performed for 20 s on each lateral face of the cylinder after the silicone mold was removed. Specimens were stored in individual vials for 48 h to complete the polymerization [20].

The specimens were evaluated under a compressive load in a universal testing machine with a crosshead speed of 1 mm/min, and the data were obtained.

### 2.5. Sorption and Solubility

Thirty disc-shaped specimens of each group (0 wt%, 10 wt%, and 16 wt% D_2_O) were fabricated using a silicone mold (6.0 mm diameter *x* 2.0 mm height) [21]. Uncured adhesive was placed in the silicon mold. A Mylar strip and a glass slide were placed onto the silicon mold, and the adhesive was light cured for 20 s. Additional light curing for 20 s was performed on the bottom of the specimen. Specimens were stored in a desiccator containing freshly dried silica gel [18]. After 24 h, the specimens were weighed using an analytical balance of 0.0001 g (Mettler Toledo, OH, USA). This cycle was repeated until a constant mass (mi) was obtained.

The specimens of each group were randomly divided into three groups (*n* = 10), according to the aging solution: water, ethanol, and acetone. Next, the specimens were immersed in 1 ml of aging solution at 37°C for 28 days. After this period, the specimens were removed, blotted dried, and reweighed (ms). The specimens were again dried inside a desiccator and weighed daily until a constant mass was achieved (md). The data were given in percentage of total loss of weight (solubility) or gain of weight (sorption) [20].

Sorption and solubility were calculated using equations (2) and (3).

### 2.6. Statistical Analysis

The obtained data of DC (%), flexural strength (MPa), modulus of elasticity (GPa), and compressive strength (MPa) were analyzed using one-way ANOVA and Tukey's test (5%). For sorption (%) and solubility (%), data were tabulated and mean values were analyzed using two-way ANOVA and Tukey's test (5%).

## 3. Results

Table 1 shows the adhesive DC, flexural strength, modulus of elasticity, and compressive strength. The results for the mechanical tests showed statistical differences among the concentrations. The adhesive model with 0 wt% D_2_O showed the lowest mean values of DC compared with the 10 wt% and 16 wt% D_2_O (*p*=0.0001). The experimental adhesive with 10 wt% D_2_O showed the highest mean values of flexural strength compared to the other groups (*p*=0.0007). For modulus of elasticity, the adhesives with 0 wt% and 10 wt% D_2_O showed higher mean values than the 16 wt% D_2_O (*p*=0.0108). Also, for compressive strength, the experimental adhesive with 16 wt% D_2_O showed the lowest mean values (*p*=0.0001).

**Figure 1:**
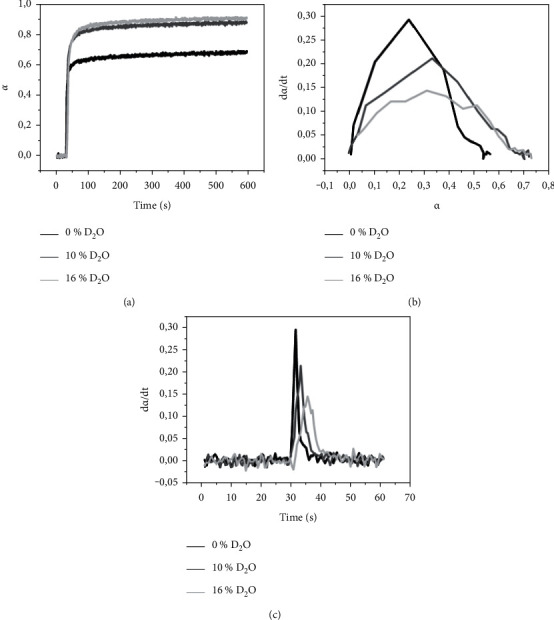
Polymerization kinetics of adhesives with different amounts of deuterium in their formulation: (a) DC over time; (b) polymerization rate by time; (c) polymerization rate by DC. *α* is the fraction of reacted monomer.

Figure 1 presents the kinetic results corresponding to the light curing of the adhesives models with different amounts of deuterium in its formulation.  Figure 1(a) presents the DC over time, and it is possible to observe an increase in conversion curve after 50 data collection, showing the light curing process from 30 to 50 s. After this initial growth, the curves show little variation over time (adhesive stability after conversion).  Figure 1(b) presents the polymerization rate over time. It is possible to observe that the effects were water dependent. These effects provided a decrease in the maximum polymerization rate, widened the peak of the derivative, and moved the maximum peak to longer times due to the increase in the amount of D_2_O.  Figure 1(c) presents the conversion rate by the DC, which means how much the reaction speed varies according to the reaction regardless of time. The adhesive with 0 wt% D_2_O had the highest conversion rates; however, this only occurred at the beginning of the reaction after the conversion of 40% (*α* = 0.4) of monomer, and there is a sharp drop in this reaction rate. The adhesives with 10 or 16 wt% D_2_O had lower conversion rates, but their decrease was slower than the 0 wt% D_2_O.

To perform a kinetic study, the autocatalytic model represented by equation (4) was used. This equation lists four constants: *k* (speed constant), *m* (exponent of the autocatalytic term), *n* (exponent of the reaction order term), and *c* (reaction yield). The autocatalytic term corresponds to the free radicals present in the polymeric chains which increase the reaction speed. The reaction order term corresponds to the double bonds reacted, and with the increase in conversion, they would decrease the reaction speed in an antagonistic way to the autocatalytic term [19].

**Figure 2:**
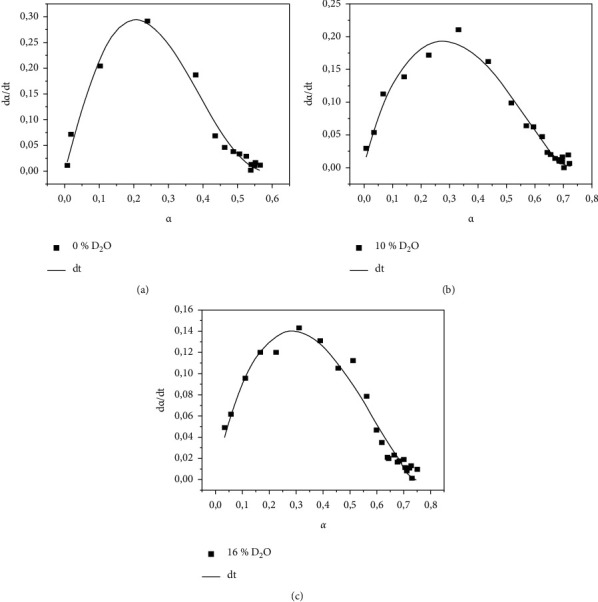
Polymerization kinetics of adhesive models with different amounts of D_2_O in their formulation.

Figure 2 presents the polymerization rate by time as a function of conversion. In the experimental data presented in Figure 2, the kinetic model for the autocatalytic reaction was adjusted, and the results obtained from this model are presented in Table 2.

The autocatalytic models had a coherent fit to the experimental values, observing an increase in velocity as a function of the yield in the first half of the reaction and a decrease in velocity from different yield values. At the beginning, the products of the reactions themselves (propagation of polymerization) stimulate the conversion, and in the second stage, the phenomenon of chain termination occurs with a decrease in the polymerization rate.

Table 2 presents the kinetic constants of the equation for autocatalytic processes (equation (4)). The results showed that the addition of D_2_O to the adhesive models caused a progressive change in the kinetic constants. As the amount of D_2_O increased, there was a reduction in the speed constant (*k*). The exponent of the autocatalytic term (*m*) did not change significantly with an increase of D_2_O in the adhesive model. The exponent of the reaction order term (*n*) was significantly reduced when comparing the 0 wt% D_2_O with the 10% and 16 wt% D_2_O, but there was no difference between the 10% and 16 wt% D_2_O. The constant *c*, which corresponds to the extent of reaction, exhibited an increase in the extent of reaction due to the increase in the amount of D_2_O in the adhesive.

Mean sorption and solubility values obtained for each group in the different aging solutions are presented in Table 3. For the sorption, water and ethanol showed statistical differences in the three adhesives' formulation (0 wt%, 10 wt%, and 16 wt% D_2_O). In acetone, experimental adhesives with 0 wt% and 10 wt% D_2_O showed significantly less mean values than the 16 wt% D_2_O. Considering the different formulations, experimental adhesive with 16 wt% D_2_O absorbed more solvent than the 0 wt% D_2_O. Among the solvents, water was the least absorbed by the samples.

Samples with 0 wt% D_2_O showed lower mean values for the solubility than the 16 wt% D_2_O in ethanol and acetone but were higher in water. Samples with 10 and 16 wt% D_2_O were not statistically different from each other, still considering the solubility in all solvents. Among all concentrations (0 wt%, 10 wt%, and 16 wt% D_2_O), ethanol showed the highest mean values for the solubility when compared with water and acetone.

**Figure 3:**
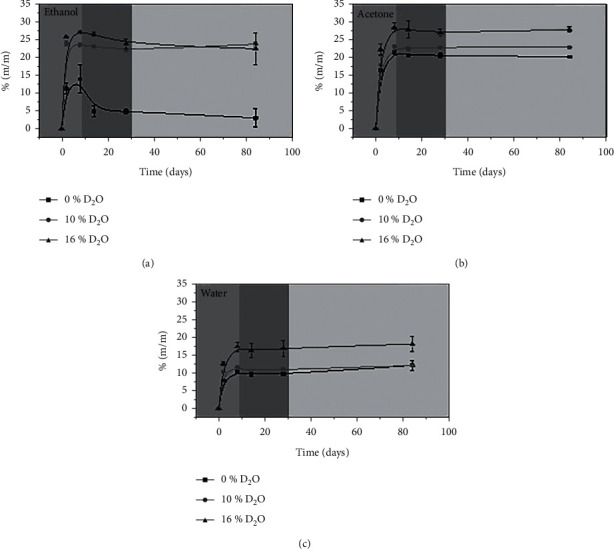
Representative graphs of the sorption of adhesives (%) in relation to the storage time.

Figure 3 presents representative graphs of the sorption of the dentin adhesive (%) in relation to the storage time. The sorption curves of adhesives with 0 wt% and 10 wt% D_2_O immersed in water and acetone showed the same characteristics. In the first two days (region A), there was an increase in mass. After that period, there was a little less mass (region B) following stabilization (region C). The adhesive with 16 wt% D_2_O immersed in water or acetone showed greater sorption, while the adhesive with 0 wt% D_2_O immersed in the same media showed less sorption.

The adhesive with 0 wt% D_2_O immersed in ethanol showed a different behavior from the others. There was an increase in mass until the second day followed by a large drop in sorption, approximately 60% of the mass absorbed in the first two days. The adhesives with 10 wt% and 16 wt% D_2_O showed, respectively, 6% and 15% loss of sorption after two days of storage (region B).

## 4. Discussion

The model adhesive resin used in the present study consisted of a mixture of HEMA and BisGMA with a mass ratio of 45/55 and photoinitiators [3]. Commercial adhesives were not used because of their unknown and complex composition that may influence results and adversely affect reproducibility [2, 21]; therefore, a fully known formulation is required to better understand the behavior of the adhesive in the wet simulated clinical environment under aging.

The photoinitiators used for the present study combine hydrophobic (0.5 wt% CQ as a hydrophobic photosensitizer and 0.5 wt% EDMAB as a hydrophobic coinitiator) and hydrophilic (1.0 wt% DPIHP hydrophilic iodonium salt) characteristics and showed good results in relation to the DC of adhesives in the presence of water as observed by previous studies [5, 18, 19]. DPIHP as a hydrophilic coinitiator improved the DC and mechanical properties of the adhesives, inducing a better behavior of the hydrophilic-rich phase [22]. This effect was related to its capacity to act as an electron acceptor, regenerating photosensitizer molecules (e.g., CQ) by replacing or generating terminating radicals. The incorporation of hydrophilic photoinitiators in the resin will allow photoinitiators to diffuse more freely within the hydrophilic-rich phases, improving their mechanical properties. However, DPIHP is inactive without the presence of a photosensitizer [22].

Different amounts of D_2_O (heavy water) were added to simulate wet bonding conditions in the dentin and to allow phase separation of the adhesive during light curing. These concentrations of D_2_O were added according to a ternary phase diagram [7], where 0 wt% D_2_O represents a neat resin, 10 wt% D_2_O is the limit of macroscopic separation for resin mixture, and in 16 wt% D_2_O, the macrophase separation occurs [5].

The polymerization reaction of an adhesive is a complex mechanism that dramatically influences its structure and properties [22, 23].

For DC, neat resin (0 wt% D_2_O) exhibited the significant lowest values and the highest rate of polymerization when compared with the formulation with D_2_O (Figures 1 and 2), as observed previously [12, 14, 24, 25]. This result could be explained due to the enhanced mobility of reactive species in the lower viscosity with the dilution in water [14, 24, 25]. The viscosity of the adhesive model with water content is lower than formulations without water [25]. The polymerization rates decrease with increasing water (D_2_O) content (Figure 2). The polymerization rate values are the result of the autoacceleration effects in free radical polymerization (gel effect), which is associated with the viscosity of the resin monomer, where higher viscosity would have a higher polymerization rate [15].

When the kinetic constants of the reactions were determined, the exponent of the autocatalytic term did not undergo a significant change by the addition of D_2_O. However, the speed constant (*k*) and the exponent of the reaction order term (*n*) decrease with the increase of D_2_O. The increase in the speed constant (*k*) caused by the absence of D_2_O may be related to the greater amount of free radicals active in the medium. Regarding the exponent of the reaction order term (n), the loss of speed due to the disappearance of the monomer is linked to a change in the reaction mechanism [18], which can be caused by the chain transfer process. However, the change in the mechanism was beneficial to the adhesive model since it allowed a greater extension of the polymerization reaction, improving its final properties.

The change of mechanism can modify the polymerization reaction speed, and the DC can influence other properties of the adhesive model, such as its interaction with water. When the initiate transfers the chain to D_2_O and starts the polymerization process, this new chain presents an additional hydroxyl when compared to the adhesive model with 0 wt% D_2_O. The increase in hydroxyl groups may favor the process of water permeability in the adhesive (hydrophilia), impacting its durability in the mouth.

The mechanical properties tested, such as the flexural strength [26], modulus of elasticity, and compressive strength [27, 28], are considerably important due to the simulation of the mechanical load during chewing. For the flexural strength, the highest mean value was observed in the 10 wt% D_2_O group, which is statistically different from the 0 wt% and the 16 wt% D_2_O (*p*=0.0319). For the modulus of elasticity, the highest mean value was also in the 10 wt% D_2_O group and was only statistically different from the 16 wt% D_2_O group (*p*=0.0179).

For the compressive strength, the samples should collapse during the application of vertical forces on the long axis of the material [29]. In the present study, only samples with 0 wt% and 10 wt% D_2_O collapsed. The specimens with 16 wt% D_2_O were accommodated according to the load increase and presented only the reduction of height and some cracks. However, even without collapsing, the test was automatically stopped by the universal testing machine.

The compressive strength showed a statistical difference among the concentrations of D_2_O in the adhesive formulation (*p* < 0.0001), where the 16 wt% D_2_O group showed the lowest values. These results can be explained because in these specimens, there are empty spaces created by the bubbles, leaving the specimens less resistant. This water content (16 wt%) exceeds the miscibility limit, causing macrophase separation of the adhesive, confirming the findings of Ye et al. [4]. Although the different water content added to the adhesive (10 wt% and 16 wt%) did not present significant differences in the propagation and termination of polymerization, the mechanical properties (compression and modulus of elasticity) were more affected with excess water (16 wt%). The different water/D_2_O concentrations that may be present in the adhesive contribute to the heterogeneity of the material, and the more heterogeneous the material, the greater the probability that a significantly weaker structure will occur in some regions. It is likely that, during the function, stress will focus on the limit of the two phases. Deterioration of the material can start at the stress concentration sites. Thus, excess water may play a critical role in the integrity and durability of the adhesive/dentin interface under stresses occurring in the oral environment.

For this reason, the first null hypothesis was rejected.

Recognizing the influence of excess water/D_2_O and types of phases allows us to better understand dental adhesives. Water is present on the dentin surface during the application of the adhesive system in different concentrations since it varies with the structure of the dentin. The sound dentin is different from the affected dentin, varying according to the depth, age of the patient, and even with the skills of the operator. In addition, water is also present in the adhesive composition (as a solvent) and extrinsically in the oral environment [23].

Sorption and solubility analyses are important physical properties to understand the performance of adhesive systems in the oral environment and in the presence of water as well as organic solvents (ethanol or acetone) present in food and beverages [30]. All adhesive formulations under aging solutions presented significant differences in the sorption behavior. All samples exhibited rapid sorption in the first 8 days of immersion (Figure 3). The highest mean values of sorption were presented in the 16 wt% D_2_O in all solutions. This result is also in agreement with Park et al. [14]. It can be explained because in the 16 wt% D_2_O, there are empty spaces where the water is entrapped due to phase separation. When this entrapped water begins to evaporate from the empty pores, they are capable of storing more solutions, accelerating degradation and reducing the mechanical properties [19].

Samples in ethanol and acetone present higher mean values of sorption than samples in water [31]. This result could be associated with the molecular weight: 18 g/mol for water, 46 g/mol for ethanol, and 58 g/mol for acetone. All adhesives' formulations under aging solutions also showed different solubility behavior. Ethanol and acetone seem to compromise the samples more than water. The solvent potential depends on the polarity between the substances [12]. The water solubility parameter is higher than the methacrylate monomers, making it a weak solvent [12, 32], which means that more time would be needed to promote greater plasticization and degradation of the polymers.

The swelling process of a polymer subjected to a medium occurs due to the osmotic effect that the polymer causes; the pure liquid has a lower vapor pressure than the liquid absorbed in the polymer [29]. The behavior of adhesives in region A (Figure 3) showed an increase in the mass of polymers. Subsequently, a loss of mass occurred (region B) due to the migration of unreacted monomers to the solution [33, 34]. The solvent penetrates the monomers' matrix and led to increased absorption and matrix plasticization, and the unreacted monomers release.

Since the solubility of monomers is greater in ethanol and acetone than in water, the results suggest the ethanol promotes greater sorption (solvent penetrates in the monomer matrix), which leads to greater solubility (hydrophilic and hydrophobic monomers' elution).

Based on the FTIR data, it can be concluded that the adhesive with 0 wt% D_2_O showed less conversion of monomers into polymers, presenting a greater amount of unreacted monomer between its chains [33, 34]. The adhesives immersed in water and acetone had a slight mass loss in region B (Figure 3) because only the HEMA has a high solubility in these media. BisGMA is soluble in ethanol; therefore, the mass loss of adhesive with 0 wt% D_2_O can be associated with the release of BisGMA to the media. The adhesives with 10 wt% and 16 wt% D_2_O did not exhibit this behavior, probably because most of the BisGMA is trapped in the chains. These results are consistent with the DC, indicating that these adhesives showed a greater extent of reaction.

Thus, the second null hypothesis was rejected.

The most effective adhesive systems have hydrophilic monomers and a high concentration of solvents, which may be water, ethanol, or acetone. The recognition and maintenance of wet dentin is difficult to achieve since it depends on the type of solvent on the adhesive as well as the skill and interpretation of the operator in relation to the manufacturer's instructions, drying time, and distance between the tooth and the syringe [35–37]. Excess water may transform the adhesive interface into a semipermeable membrane, highly susceptible to the degrading effects of water [38].

Researchers have established that water-based and water/ethanol-based adhesives have better performance on dry surfaces (such as in endodontic treated teeth) and acetone-based adhesives have better performance on wet surfaces [39]. However, from the clinical point of view, it is practically impossible to accurately determine the ideal surface moisture. Furthermore, the presence of water in the hybrid layer may compromise the formation of a highly cross-linked polymer, making the wetting technique difficult and unpredictable [6, 40].

Some investigations show that the bonding of dry demineralized dentin may be an option to reduce the amount of water trapped inside the hybrid layer and the problems of this excess water. Reis et al. [37] observed that it is possible to obtain high bond strength values between the adhesive and air-dried dentin. The friction action can increase component kinetics and allow better diffusion of monomers inward while solvents spread out [34]. Zander-Grande et al. [40] clinically evaluated restorations with adhesion in dry and wet dentin, and the authors concluded that dentin hydration does not have a significant effect on adhesive retention, provided the clinician's vigorously friction action with the adhesives on the dentin surfaces [40].

With the understanding that excess water is extremely detrimental to the different properties of the adhesive, it is necessary to perform more research on the behavior of adhesive systems under the most diverse clinical conditions since it will be subject to excess water in dentin and lack of water in endodontic treated teeth; hypermineralized and whitened dentin; young or old; and healthy, caries-affected, or fractured. The discovery of new components with better hydrophobic and hydrophilic characteristics is also possible.

## 5. Conclusion

The different concentrations of water (D_2_O) added in the experimental adhesive formulation and aging solvents significantly influence the physicochemical and mechanical properties of the experimental model. The 10 wt% of water might have positively influenced the degree of conversion, flexural strength, modulus of elasticity, compressive strength, and sorption and solubility of the dentin adhesive model, where excess and lack of D_2_O were harmful to the adhesive [40].

Degree of conversion:

(1) $$\begin{array}{c} DC\left(\%\right)=\left(1-\left(\frac{\left(1638cm-1/1608cm-1\right)\;cured}{\left(1638cm-1/1608cm-1\right)\;uncured}\right)\right)\times \;100. \end{array}$$

Sorption:

(2) $$\begin{array}{c} WS\;\left(\%\right)=\left(\frac{m_{s}-m_{i}}{m_{i}}\right)x100. \end{array}$$

Solubility:

(3) $$\begin{array}{c} WL\;\left(\%\right)=\left(\frac{m_{i}-m_{d}}{m_{i}}\right)x100. \end{array}$$

Autocatalytic processes:

(4) $$\begin{array}{c} \left(\frac{d\alpha }{dt}=k{\left(\alpha \right)}^{m}{\left(c-\alpha \right)}^{n}\right). \end{array}$$

## Figures and Tables

**Table 1:** Mean ± standard deviation and results of Tukey's test of degree of conversion, flexural strength, modulus of elasticity, and compressive strength of adhesives.

	Degree of conversion (%)	Flexural strength (MPa)	Modulus of elasticity (GPa)	Compressive strength (MPa)
0 wt%	68.09 ± 0.14 A	109.4 ± 20.5 A	2.7 ± 0.2 A	141.4 ± 19.5 A
10 wt%	87.07 ± 0.81 B	141.6 ± 6.71 B	2.7 ± 0.4 A	151.2 ± 27.8 A
16 wt%	89.87 ± 0.24 B	107.8 ± 15.8 A	2.2 ± 0.3 B	98.8 ± 18.0 B

Different letters represent statistically significant differences (*p* < 0.05). Uppercase letters refer to columns.

**Table 2:** Kinetic constants referring to the autocatalytic model adjusted to the experimental results presented in Figure 2.

Adhesive models	*k*	*m*	*n*	*c*
0 wt% D_2_O	15 ± 9	1.17 ± 0.17	2.11 ± 0.47	0.58 ± 0.03
10 wt% D_2_O	2.01 ± 0.53	0.9 ± 0.1	1.41 ± 0.2	0.71 ± 0.02
16 wt% D_2_O	1.27 ± 0.32	0.88 ± 0.1	1.42 ± 0.2	0.74 ± 0.02

*k*, speed constant; *m*, exponent of the autocatalytic term; *n*, exponent of the reaction order term; *c*, reaction yield.

**Table 3:** Mean ± standard deviation and results of Tukey's test for sorption (% SOR) and solubility (% SOL) for each experimental group.

	% SOR			% SOL		
	Water	Ethanol	Acetone	Water	Ethanol	Acetone
0 wt%	8.6 ± 0.2 Aa	20.8 ± 0.4 Ab	21.3 ± 1.7 Ab	2.1 ± 0.7 Aa	5.6 ± 1.9 Ab	0.2 ± 0.4 Ac
10 wt%	7.2 ± 0.3 Ba	19.7 ± 0.5 Bb	22.5 ± 0.3 Ac	0.4 ± 0.2 Ba	7.0 ± 0.4 ABb	4.7 ± 1.2 Bc
16 wt%	11.7 ± 0.4 Ca	24.9 ± 0.7 Cb	27.8 ± 1.2 Bc	0.2 ± 0.1 Ba	7.6 ± 0.4 Bb	3.3 ± 2.2 Bc

Different letters represent statistically significant differences (*p* < 0.05). Uppercase letters refer to columns; lowercase letters refer to lines.

## Data Availability

The data used to support the findings of this study are available from the corresponding author upon request.
